# INCIDENCE OF ACUTE TRAUMA ON HAND AND WRIST: A RETROSPECTIVE STUDY

**DOI:** 10.1590/1413-785220172506169618

**Published:** 2017

**Authors:** GIOVANNA DAMM RAPHAEL JUNQUEIRA, ANDRÉ LUIZ MACHADO LIMA, ROBISON BONI, JOELMAR CÉSAR DE ALMEIDA, RAFAEL SOUZA RIBEIRO, LEANDRO AZEVEDO DE FIGUEIREDO

**Affiliations:** 1. Orthopedics and Traumatology Service, Santa Casa de Misericórdia de Vitória Hospital, Vitória, ES, Brazil.; 2. Hand Group, Orthopedics and Traumatology Service, Hospital Estadual Doutor Jayme dos Santos Neves, Serra, ES, Brazil.

**Keywords:** Hand injuries/epidemiology, Wrist injuries/epidemiology, Emergency medical services, Orthopedics., Traumatismos da mão/epidemiologia, Traumatismos do punho/epidemiologia, Serviços médicos de emergência, Ortopedia.

## Abstract

**Objectives:**

A retrospective statistical data gathering of wrist and hand complaints assisted over two years in the orthopedic emergency department of a regional referral hospital, seeking to know the profile of these patients.

**Methods:**

Information obtained by analysis of 31.356 orthopedic visits from May 2013 to April 2015, of which 6.754 related to hand complaints and/or wrist, at the Hospital Estadual Doutor Jayme dos Santos Neves (HDJSN) and analyzed by IBM SPSS Statistics software version 21.

**Results:**

The data revealed that the average age was 37,5 ± 15,7 years and the male gender was predominant (60,72%). Bruises (52,58%) and fractures (30,49%) were the most common diagnoses.

**Conclusion:**

The complaints of wrist and hand accounted for 21,44% of all orthopedic emergency room visits. Detailed data description and correct definition of the International Classification of Diseases (ICD-10) are needed to better define the epidemiological profile of patients seeking orthopedic emergency. **Level of Evidence III, Retrospective Study.**

## RESUMO

**Objetivos:**

Fazer um levantamento de dados estatísticos retrospectivos dos atendimentos de lesões de punho e mão, ao longo de dois anos no pronto-socorro ortopédico de um hospital de referência regional, visando conhecer o perfil desses pacientes.

**Métodos:**

Informações obtidas por análise de 31.356 atendimentos ortopédicos no Hospital Estadual Doutor Jayme dos Santos Neves (HDJSN) entre maio de 2013 e abril de 2015, dos quais 6.754 apresentaram lesões na mão e/ou punho. Os dados foram analisados pelo programa IBM SPSS Statistics versão 21.

**Resultados:**

Os dados revelaram que a média de idade foi de 37,5 ± 15,7 anos, com predominância do sexo masculino (60,72%). Contusões (52,58%) e fraturas (30,49%) foram os diagnósticos mais frequentes.

**Conclusão:**

As lesões do punho e da mão corresponderam a 21,44% do total de atendimentos ortopédicos de emergência. A descrição detalhada dos dados e a definição exata na Classificação Estatística Internacional de Doenças e Problemas Relacionados com a Saúde (CID-10) são necessárias para determinar melhor o perfil epidemiológico do paciente que procura a emergência ortopédica. **Nível de Evidência III, Estudo Retrospectivo.**

## INTRODUCTION

Acute traumas involving upper limb in the emergency room are common, however, they are little understood from an epidemiological perspective.^1^ The injuries that affect the distal extremity of the upper limb are considered a major social and public health problem both due to the physical and mental impact, as well as to high costs of initial treatment of its sequels.^2,3^ According to the *National Eletronic Injury Surveillance System* (NEISS), lacerations and fractures of the fingers and hands are the anatomical sites most affected in the work accidents attended in the American emergency services.^1^ It is estimated that approximately 11-20% of visits to emergency departments in the United States are due to injuries to the hands and wrists, making the epidemiological analysis of these lesions of paramount importance.^3^ ^-^ ^5^ It is known that the costs of falling productivity due to absence from work , in general, are more expensive than the treatment of the injury itself.^3^ When added, the costs of absence from work with medical and hospital expenses can reach an average of thirty thousand dollars per injury.^6^ The social and economic costs cannot be measured only by the social security aspect, for not expressing its real dimension. The issue becomes more important if we consider, for example, the cost of specialized medical care, with more complex procedures, the drop in production resulting from absenteeism and the functional reduction consequent to the possible sequelae.^7^ The purpose of this study is to evaluate the characteristics of the injuries as well as to calculate epidemiological estimates of the traumatic injuries that affect the hand and the wrist by means of a population sample of patients attended in the orthopedic emergency of a reference hospital in trauma during two years, in the state of Espírito Santo. The hand would be the terminal segment of the upper limb, continuation of the fist, ending distally with the fingers. Its proximal limit would be given by a horizontal plane that passes through the pisiforme and the scaphoid. Its skeleton would correspond to the second row of the carpus (trapezoid, trapezoid, capitate and hamato), metacarpal bones and phalanges. The first row (scaphoid, lunate, pyramidal and pisiform) along with the distal end of the radius and ulna would belong to the wrist region.

## MATERIALS AND METHODS

This is a cross-sectional epidemiological study. All information was obtained by means of data collection in the medical records of the orthopedic emergency room, defining the complaints concerning the wrist and the hand. Trauma denominations in these regions were classified according to the International Code of Diseases (ICD-10) and individual assessment of medical records. The study will cover visits between May 2013 and April 2015.

All researches used as a bibliographic source were collected using search sites such as PubMed and ClinicalKey, using the keywords “injury”, “wrist”, “hand”, “emergency”, “epidemiology” and “trauma”.

### Calculation of rate:

The sample size was 6,767. The variables analyzed in the wrist and hand traumas were: gender, color, age, municipality of origin and affection.

The project of this research was approved by the Ethics Committee (CAAE 50648015.1.0000.5065 ) of the Superior School of Sciences of Santa Casa de Misericórdia de Vitória on March 29, 2016.

The program used in the analyzes was the IBM SPSS Statistics version 23.

The data characterization was performed through the observed frequency, percentage, minimum, maximum, mean and standard deviation.

The Chi-square test verified the association between qualitative variables. To compare quantitative and qualitative variables, variance analysis (ANOVA) was used using Dunnett’s multiple comparison test, since the variances were not homogeneous (Levene’s test).

The level of significance adopted in all analyzes was 5% with a 95% confidence interval.

## RESULTS

Between May 2013 and April 2015, there were 101,769 visits to the emergency room of the reference trauma hospital in the city, orthopedic visits were 31,718, which is the specialty with the highest number of records, followed by the medical clinic, with 30,207 And general surgery, with 26,212.

Of the orthopedic visits, 21.6% were of complaints related to wrist and hand. Even though this number is relatively large, corresponding to around 282 calls per month, this number is known to be far from realistic. Many of the injuries of the wrist and hand give entry to the PS for other specialties (mainly of the general surgery), it is up to the orthopedist to respond only to the opinion requested by the surgeon, thus keeping the record of the service as general surgery.

The present study showed that the orthopedic care of all the visits in the emergency room of the reference unit in trauma during the period evaluated corresponded to 31.2%. (Table 1)

**Table 1:** Orthopedic PS attendances.

	n	%
Orthopedic attendances	31718	31.2
Other attendances	70051	68.8
Total	101769	100.0

Source: Data from PS records.

Among the orthopedic visits, 21.6% corresponded to the complaints due to complaints in the distal regions of the upper limb, demonstrated in this work by the wrist or hand. (Table 2)

**Table 2:** Wrist/hand complaints at the orthopedic PS.

	n	%
Wrist and hand complaints at the orthopedic PS	6767	21.6
Other orthopedic attendances	24588	78.4
Total	31355	100.0

Fonte: Data from orthopedic PS records.

The male gender corresponded to 60.7% of the total sample. The parda color obtained 46.3%. The municipality of Serra was the one that presented the highest proportion of attendances with 90.7%, in which, together with the other municipalities of Grande Vitória, in addition, they presented 97.8% of the origin of those served, 1.8% referring to other municipalities of the state and 0.5% to municipalities in other states. The monthly distribution of attendances maintained a similar absolute value, varying from 512 (December months) to 592 (October months). The minimum age was 7 years, maximum of 99 years, an average age of 37.5 years and standard deviation of ± 15.7 years. (Table 3)

**Table 3:** Demographic characterization.

		n	%
Gender	Male	4109	60.7
	Female	2657	39.3
Color	Pardo color	3141	46.4
	White	1440	21.3
	Black	596	8.8
	Yellow/Indigene	105	1.6
	No Information	1485	21.9
Procedence	Grande Vitória	6616	97.8
	Other municipalities of ES	119	1.8
	Outher States	32	0.5
Monthly Distribution	January	564	8.3
	February	529	7.8
	March	591	8.7
	April	524	7.7
	May	576	8.5
	June	556	8.2
	July	576	8.5
	August	589	8.7
	September	589	8.7
	October	592	8.7
	November	569	8.4
	December	512	7.6
Age	Minimum	Maximum	Medium
	7.0	99.0	37.5 (± 15.7)

Source: Data from orthopedic PS records.

Regarding the affection, Contusion (52.5%) and Fracture / Dislocation (34.3%) had higher percentages. (Table 4)

**Table 4:** Affection.

	n	%
Contusion	3555	52.5
Fracture/dislocation	2322	34.3
Short injury content	444	6.6
Pain	268	4.0
Tenosynovitis	68	1.0
Amputation	60	0.9
Convalescence	31	0.5
Infection	19	0.3
Total	6767	100.0

Source: Data from orthopedic PS records.

The topographic distribution of the complaints was done as follows: Fist (40.8%), Fingers (32.0%) and Hand (22.8%). Regions that were not specified in the medical records accounted for 4.4% of complaints. (Table 5)

**Table 5:** Topographic Distribution of the complaints.

	n	%
Wrist	2763	40.8
Finger	2164	32.0
Hand	1545	22.8
Not specified	295	4.4
Total	6767	100.0

Source: Data from othopedic PS records.

**Figure 1:**
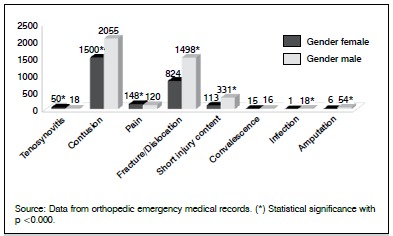
Disorders distributed by gender.

A significant association of gender with affection (value / p = 0.000) was observed, and the positive contribution to significance occurred in the male gender with amputation, short-blunt injury, fracture / dislocation, and infection. In the female gender, positive significance occurred with contusion, pain and tenosynovitis. (Figure 1)

**Figure 2:**
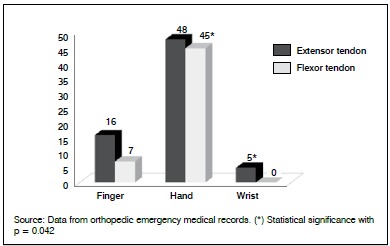
Tendon lesions according to topographic distribution.

The association between topographic distribution and tendinous lesions was significant (p value = 0.042). The lesions, when they occurred in the central region of the Hand, had a statistically significant relationship with lesions of the flexor tendons. The extensor lesions were positively associated with wrist injuries. (Figure 2)

## DISCUSSION

In a study carried out in a university hospital in Ribeirão Preto, an analysis of the demand for emergency care was made in 2000, in which 27.6% corresponded to traumatic injuries involving the hands.^6^ Comparing the data obtained in this study with the literature, a strong predominance of the male gender was observed, with 60.7%. The data of this work presented results similar to those of Santos et al.,^8^ Lopes^9^ and Batista and Filgueira.^10^ The results found are in accordance with clinical experience, since men are more exposed to the risk of accidents, men in this way were responsible for more severe trauma records, such as amputation, short-blunt injury, fracture / dislocation and infection. On the other hand, women with 39.3% presented trauma considered milder, such as bruising, pain and tenosynovitis, and were statistically significant in both cases.

 Accidents related to work tasks include, mainly, trauma and short bruised wounds on the hand, wrist and head, along with eye injuries. More intensive supervision in the use of protective equipment, more appropriate training in risk recognition, and safe working practices, including vehicle operation in the workplace, should be implemented to reduce work-related injuries.^11^

A 2009 consultation by the National System of Electronic Surveillance (NEISS) resulted in 92,601 records of upper extremity lesions treated in an emergency department in the US in 2009, which translates into an estimated total of 3,468,996 such injuries that year. This corresponds to an incidence of 1,130 upper extremity lesions per 100,000 population per year.^12^

It was observed in this study that the incidence of flexor tendon injuries is greater when compared to extensor tendon injuries, most of them in the palm region, while extensor injuries affected the wrist region more, according to data from the literature. These lesions are usually associated with nerve damage. This is usually due to the hand-inflicted mechanism of trauma (often a knife or glass) that contains many delicate anatomical structures in the vicinity (superficial and deep flexor tendons, Joint ligament, arteries, and nerves)^13^ which are often not reported in the ICD-10 registry, so despite the effectiveness of the computerization of care, and the mandatory registration of ICD-10 to initiate care, many times the code may not correspond with actual patient injury.

This study raised the data of the emergency room visits during two years, stratifying the orthopedic care, and showing that more than 1/5 of all care is related to hand and wrist trauma, with a great impact on the volume of care delivered for orthopedics. Based on this survey, other studies may be designed with a view to reducing trauma-related accidents on the hand, or even the need for specialized care by a hand surgeon in the initial evaluation of the patient.

## CONCLUSIONS

Among all those attended, the male gender and the parda race had a higher prevalence. 

The most prevalent affections were contusion and fractures, with the hand being the region most affected.

Wrist and hand conditions accounted for 21.44% and all orthopedic care in the HDJSN emergency room between May 2013 and April 2015.

Standardized information and registration methods are essential for data to be compared.

The results of previous studies in the area of hand and wrist injury may be comparable in some areas, but differences may occur due to variations in methods of data recording and classification.

Better utilization of the international disease code (ICD-10), with accurate injury record, would facilitate and standardize the searches and documentation of patients with trauma to the wrist and hands.
